# Nutrition-related non-communicable disease and sugar-sweetened beverage policies: a landscape analysis in Kenya

**DOI:** 10.1080/16549716.2021.1902659

**Published:** 2021-04-20

**Authors:** Milkah N Wanjohi, Ann Marie Thow, Safura Abdool Karim, Gershim Asiki, Agnes Erzse, Shukri F Mohamed, Hermann Pythagore Pierre Donfouet, Pamela A Juma, Karen J Hofman

**Affiliations:** a Maternal and Child Well Being Unit, African Population and Health Research Center, Nairobi, Kenya; b Menzies Centre for Health Policy and Director of Academic Titles, School of Public Health, The University of Sydney, Sydney, Australia; c SAMRC Centre for Health Economics and Decision Science Research-PRICELESS SA, School of Public Health, University of the Witwatersrand, Johannesburg, South Africa; d Health and Systems for Health Unit, African Population and Health Research Center, Nairobi, Kenya; Academic Unit of Primary Care (AUPC) and the NIHR Global Health Research Unit on Improving Health in Slums, University of Warwick, Coventry, UK; e Lown Scholars Program, Department of Global Health and Population, Harvard T.H. Chan School of Public Health, Boston, MA, USA; f Social Policy Panning Monitoring and Evaluation (SPPME), UNICEF, N'Djamena, Tchad; g Independent Research Consultant; h University of the Witwatersrand, Johannesburg, South Africa

**Keywords:** Diet, politics, actors, industry tactics, data review, policy review

## Abstract

**Background**: The burden of undernutrition is significant in Kenya. Obesity and related non-communicable diseases are also on the increase. Government action to prevent non-communicable diseases is critical. Taxation of sugar-sweetened beverages has been identified as an effective mechanism to address nutrition-related non-communicable diseases, although Kenya is not yet committed to this.

**Objective**: To assess the policy and stakeholder landscape relevant to nutrition related non -communicable diseases and sugar-sweetened beverage taxation in Kenya.

**Methods**: A desk review of evidence and policies related to nutrition related non-communicable diseases and sugar-sweetened beverages was conducted. Data extraction matrices were used for analysis. Key informant interviews were conducted with 10 policy actors. Interviews were thematically analysed to identify enablers of, and barriers to, policy change towards nutrition-sweetened beverage taxation.

**Results**: Although nutrition related non-communicable diseases are recognised as a growing problem in Kenya most food-related policies focus on undernutrition and food security, while underplaying the role of nutrition related non-communicable diseases. Policy development on communicable diseases is multi-sectoral, but implementation is biased towards curative rather than preventive services. An excise tax is charged on soft drinks, but is not specific to sugar-sweetened beverages. Government has competing roles: advocating for industrial growth, such as sugar and food processing industries to foster economic development, yet wanting to control nutrition related non-communicable diseases. There is no national consensus about the dangers posed by sugar-sweetened beverages.

**Conclusion**: Nutrition related non-communicable diseases policies should reflect a continuum of issues, from undernutrition to food security, nutrition transition, and the escalation of nutrition related non-communicable diseases. A local advocacy case for sugar-sweetened beverage taxation has not been made. Public and policy maker education is critical to challenge the prevailing attitudes towards sugar-sweetened beverages and the western diet.

## Background

Undernutrition and food insecurity have historically been, and remain, significant public health problems in Kenya [1,2]. Consequently, there are many policies addressing these issues but few include actions to address unhealthy diet and over-nutrition. A policy gap has emerged due to the nutrition transition related to increasing urbanisation. Kenya is experiencing increasing levels of obesity and related non-communicable diseases (NCDs), including diabetes, cardiovascular diseases and cancer [3–5]. Nutrition related-NCDs (NR-NCDs) are higher among urban than rural communities, and women are particularly vulnerable [3,4,6]. For instance, obesity among women increased by almost 10-fold (25% to 33%) from 2008 to 2014 [5]. An estimated 4% of rural and 21% of Kenyan urban children are overweight/obese [5]. Furthermore, NCDs account for about one third of all deaths and half of all hospital admissions in the country [6]. Kenya’s health policy and the national strategy for the prevention and control of NCDs (2015–2020) emphasize the need for action to reverse and address the NCD burden [7,8].

The World Health Organization (WHO) recommends the taxation of sugar-sweetened beverages (SSBs) as a population-level and cost-effective intervention to control the rising burden of obesity and NR-NCDs [9]. SSBs refer to any beverage with added sugar or other sweetener, such as sucrose or high fructose corn syrup, which have high levels of calories and little nutritional value [10]. Examples of SSBs include regular soda, fruit drinks, sports drinks, energy drinks, sweetened waters, and coffee and tea beverages with added sugars.

Fiscal interventions such as SSB taxes have the potential to not only reduce obesity and related NCDs, but to also raise additional revenue, which can be further directed towards health promotion or health care delivery [11] for prevention and management of NCDs.

Despite their low-cost and efficacy, the adoption and implementation of such policies in Kenya has not occurred. An analysis of NCD prevention policies in Kenya, conducted in 2017, showed that, although some policies addressing NR-NCDs existed, they did not adequately reflect the *‘best buy interventions’* for unhealthy foods [4]. Previous analyses of the policy landscape in Kenya identified the lack of political commitment and inadequate resources in government as hampering the implementation and adoption of comprehensive NCD prevention policies [4]. The political economy of fiscal measures and ensuing tension between Government and industry is complex [12]. Kenya is one the world’s largest producers of tea and hot drink, which constitute the largest segment of the non-alcoholic drinks market. Coca Cola has a strong presence in the country and is consumed by approximately half the population [13]. Locally, there is a robust network of financial and human resources, and a growing global experience of tactics, that can be used against over-burdened and under-resourced Government departments when industry profits are threatened [14,15]. The Government’s political and governance environment is subject to resource and capacity constraints [4].

This study addresses the policy and stakeholder environments influencing NR-NCD and SSB action in Kenya. It identifies gaps in the evidence and data, and provides an analysis of the policy context and nature of the SSB industry in Kenya. Understanding the broader NCD policy landscape and the relevant politico-economic factors that impact on policy-making is a key step in the development of feasible and context-specific policy and action against SSBs.

## Methods

### Study design

This study was nested in a broader study of seven countries in East and Southern Africa, which shared the same methodology [16]. The study design was a qualitative policy analysis, which entailed a desk review of existing nutrition-related policies and evidence related to NCDs and SSBs in Kenya. We reviewed Kenyan 15 policy documents across sectors implicated in NCD prevention and SSB taxation, including documents from the departments of health, agriculture and finance.

The desk review was complemented by interviews with 10 knowledgeable policy actors from government ministries, with responsibilities related to fiscal policy and/or nutrition, health; civil society organisations with interests in NR-NCDs and SSB, industrial associations and academics (Table 1). The semi-structured interviews were designed to explore the policy and political context, as well as to identify enablers of, and barriers to, policy change with regard to NR-NCDs and SSBs. Organisations were purposively selected from the different sectors, based on the document review and the likely roles of their institutions in the formulation and implementation of NCD prevention policies and SSB taxation. Snowball sampling was then used to identify potential respondents within the selected institutions, who were formally invited to participate.

**Table 1.** Key informant interviews by organisation type and sectoral interest

*Type of organization*	*Main sectoral interest*	*Number of*
National Government	Ministry of Health: Nutrition	1
	Ministry of Health: NCDs	1
	Ministry of Health: Regulations and standards	2
Civil society and non-governmental organisations	NCDs	2
	Legal	1
	Finance/economy	1
Academia	Health	1
Industry and industry associations	Industry association	1
Total		10

The interviews were conducted using an open-ended interview guide that was tailored to the expertise of the respondent, in order to understand the gaps and opportunities to strengthen NR-NCD and SSB tax-related policies. It also included the potential of policies regarding the use of revenue to increase access to healthy food and the barriers to and facilitators of policy reforms on the same. Interviews were conducted in English and were audio-recorded with the respondent’s consent.

### Data processing and analysis

Interviews were transcribed verbatim, anonymised, and stored in digital format. They were coded manually, by two researchers, using pre-determined data matrices [16]. An iterative approach was adopted in the analysis of data from the desk review and from the qualitative interviews. Kingdon’s framework was used to draw together data sources that provided an understanding of the ‘problem’ of NCDs and SSBs, the ‘solutions’ (including an SSB tax), the existing policy landscape, and stakeholder politics [17].

### Ethical considerations

The study was approved by the Amref Health Africa – Ethical and Scientific Review Committee (Amref-ESRC), Ethics number: P593/2019. Written informed consent to participate, and consent to record the interviews, was obtained from all respondents. Confidentiality was assured and maintained.

## Results

Based on the Kingdon’s framework [17], the study findings are presented in three thematic areas: i) the availability of evidence and understanding of NCDs and SSBs as a problem in Kenya; ii) the policy content relevant to NCDs and SSBs; iii) the stakeholder politics around NCDs and SSBs, as well as the facilitators of, and barriers to, the adoption of an SSB tax (Table 2).

**Table 2.** Summary of findings using three categories used in Kingdon’s theory of agenda setting []

Kingdon Category	Facilitators	Barriers
*Category 1*: *The availability of evidence and understanding of NCDs and SSBs as a problem*	The NCD epidemic is understood to be a growing problem, driven by the nutrition transition and the increasing adoption of western diets.	Gaps in data relating to SSBs. Cultural acceptability of SSBs is a barrier to fostering political will for actions for SSB taxation.
*Category 2*: *Policies on NR-NCDs and SSBs*	Multi-sectoral policies addressing food insecurity and malnutrition present an opportunity to integrate overnutrition and NCDs into existing policy. There is excise tax on soft drinks but not for SSBs, specifically.	Policies and resources currently prioritise treatment and management of NCDs, and less of NCD prevention
*Category 3*: *Stakeholder politics related to NR-NCDs and SSB*	Civil society and the Ministry of health actively lobbied Government Treasury in 2016 to adopt SSB taxation	There is no national consensus on dangers posed by SSBs. The SSB industry is organised and actively opposes SSB taxation.

### Category 1: the availability of evidence and understanding of NCDs and SSBs as a problem

Evidence is available on the prevalence of NR-NCDs in Kenya. A nationally representative survey on NCDs prevalence and risk factors in adults was conducted in Kenya in 2015, which covered cardiovascular diseases (CVD), and their risk factors including consumption of salt, fat, sugar and alcohol in the adult population [6]. Food consumption data and nutrition indicators, focusing mainly on dietary diversity and adequacy, in women and children and not men, are periodically collected through the demographic health survey (DHS) in Kenya. The most recent DHS was conducted in 2014 [18]. Regular population level surveillance related to under-nutrition (wasting, stunting, underweight, dietary diversity, dietary adequacy) and associated risk factors is conducted at regional levels for vulnerable populations, e.g. children younger than 5 years.

Despite the existing data, respondents felt that, there was still a deficit of evidence that hampered the monitoring of NCDs and, subsequently, NCD programmes and interventions in the country. For example, no local data on the potential public health impact of SSB taxation had been collected.

There is a dearth of publicly available information on SSB sales and consumption in Kenya. However, the average annual national sugar consumption was estimated to be slightly less than 800,000 metric tons from 2008 to 2013. In 2018/2019, a 30% increase in sugar production in Kenya was forecast, as well as an increase in sugar consumption, as a consequence of the growth of the retail, industrial and food service sectors [19]. The country does not produce refined sugar and imports this for the industrial and food and beverage industries. In 2018, about 280,000 metric tonnes of sugar was imported into the country, an increase from about 190,000 in 2014 [19]. The sugar industry, directly or indirectly, supports about 6 million Kenyans, representing about 6% of the national population, and contributes 7.5% of the country’s gross domestic product [20].

Some annual industry reports on SSB production are available but these are not routinely accessible on the SSB company websites. Accessible industry information in the media lacks detailed information on SSB sales and consumption rates. However, data purchased from Fitch solutions showed that spending on non-alcoholic drinks increased in Kenya in 2019 [13]. Inadequate information about the SSB industry was identified by respondents as a barrier to the introduction of an SSB tax. Some felt that the available evidence about the SSB industry was not sufficient to convince policy makers to tax SSBs.

‘There’s quite a bit that has been done on alcohol and tobacco, we need to work on the sugar-sweetened beverages. I am not sure we have put together enough evidence [on SSB], that’s an area we can improve.’ KII- representative from civil society 3

Health sector policies recognise NCDs as a growing problem; unhealthy diets are understood as a major risk factor. For example, the Heath Policy 2012–2030 acknowledges unhealthy diets and obesity as major risk factors for poor health and states that diabetes is among the 10 major causes of mortality and morbidity in the country [7]. NCDs were viewed as a rapidly growing problem in Kenya. Respondents highlighted that, in the past, NCDs were perceived to be a problem of the affluent, but now occur in low income settings. Unhealthy dietary practices, such as consumption of calorie-dense staple foods and poor intake of fruits and vegetables, over-consumption of sugar and high salt intake, especially in processed food, were identified by respondents as major causes of NCDs. They attributed the consumption of unhealthy foods to poor information about healthy food, nutrition transition, and aspirational attitudes towards western diets, especially in urban environments.

The problem with Kenyans … is that we have a nutritional transition problem, there is a lot of pressure from the global trading community, especially amongst the youth to push them towards high sugar content drinks and foods with high fat and high salt content.” KII -representative from civil society, 1.

The ‘*National Food and Nutrition Security Policy’* explicitly implicates the nutrition transition and the shift from traditional foods, low in fat and fibre, to commercially-processed foods, as a reason for the increasing prevalence of NCDs [21].

n the past, Kenyan communities consumed foods that are low in fat and rich in fibre. Recently, however, diet related non-communicable diseases (NCDs) … have been on the increase. Contributing factors are diverse and include imports and local production of more processed foods, changes in life-style, eating habits, urbanization and globalization. … This “nutrition transition” has been growing in Kenya for some time and there is now a noted increase in the prevalence of NCDs (National Food and Nutrition Security Policy)[^1^,p.31].

Although health sector policy documents, such as the NCD strategy, generally mentioned high consumption of sugar as one component of unhealthy diets, they were not explicit about the role of SSBs as a risk factor for NCDs. Respondents felt that SSBs presented more of a challenge to health promoters than tobacco and alcohol, as there was a knowledge deficit regarding the unhealthiness of SSBs in the general population and, hence, a greater social acceptance of SSBs.

An important barrier when we are dealing with SSBs is the fact that the public does not appreciate that this (SSB consumption) is a problem. … What do respectable old men and women in the village, church elder’s take when they go for a meeting, and it is tea, coca cola, sprite, Fanta. It presents a subtle challenge that we don’t have when we are dealing with, tobacco nobody argues about the adverse consequences of tobacco or of alcohol.” KII- representative from Ministry of Health, 4.

### Category 2: policies on NR-NCDs and SSBs

The reviewed policies (Table 3) recognised the importance of diet and access to adequate food for the Kenyan population. There were strong statements about the need to prevent NCDs in policies emanating from the health sector, such as the Kenya Health policy [7]). National *‘whole of government’* policy documents, including the Constitution of Kenya 2010 and the National Development plan (Vision 2030), highlight the right of Kenyans to adequate food of acceptable quality, and the highest attainable standard of health and universal health coverage, but do not address NCDs directly [22,23].

**Table 3.** Kenyan policy documents related to NR-NCD

POLICY TYPE	DOCUMENT	OBJECTIVE RELEVANT TO NUTRITION AND DIET	ACTORS
Broader policy environment	Kenya Constitution	Highlights the right of Kenyans to adequate food of acceptable quality	Government of Kenya*
	Vision 2030	Highlights the right of Kenyans to adequate food of acceptable quality	Government of Kenya*
	Big 4 Agenda	Represents the Government’s four priority areas (pillars) during the 2017–2022 period. Food and nutrition security is one of the pillars, but focus is on food production and provision. Industrialisation is also one of the priority areas.	Government of Kenya*
	Kenya Heath Policy 2012–2030	Halt and reverse the rising burden of NCDs. To be achieved by addressing major risk factors, including unhealthy diets	Ministry of Health, stakeholders in health, development, and implementing partners
	Health Sector Strategic and Investment Plan 2013–2018	Halt and reverse rising burden of NCDs	Government of Kenya*
	Food and Nutrition Security Policy (FNSP) 2011	Achieve adequate nutrition for optimum health of all Kenyans; increase the quantity and quality of food available, accessible and affordable to all Kenyans	Government of Kenya*
NCD related policies	National Strategy for the Prevention and Control of NCDs 2015/20	Objective 3 focuses on promoting healthy lifestyles and implementing interventions to reduce modifiable risk factors for NCDs, and recommends implementation of health-related legislations and regulations on salt, saturated and trans fatty acids and refined sugar content of processed foods, and packaging, labeling and marketing of food products and beverages	Ministry of Health, academic and research institutions, NGOs, civil society, patient support groups
Nutrition and diet related policy documents	National Nutrition Action Plan 2012–2017	Objectives are to improve: (a) prevention, management and control of diet-related NCDs; (b) promote nutrition in schools, public and private institutions; and (c) nutrition knowledge, attitudes and practices among the population	Ministry of Health, Ministries of Agriculture, Education, Gender and Social Services, Planning and National Development, Medical Services, Northern Kenya and Other Arid Lands, academic and research institutions, NGOs, civil society, implementing and development partners
	National Guidelines for Healthy Diets and Physical Activity 2017	Objective 1: Provide principles of healthy diets for the general population; Objective 2: Establish a set of dietary guidelines for the Kenyan population throughout the life cycle; -promotes lower consumption of sweetened food/drinks and higher consumption of fruit/vegetables	Ministry of Health, Division of Non-communicable Diseases and Ministry of Agriculture Livestock and Fisheries. The committees and technical working groups in the Nutrition and Dietetics Unit (NDU), food and nutrition linkages, implementing, academic and research institutions, implementing partners and development partners
	School Health Policy (2017)	Thematic areas call for provision of diverse, safe and nutritious food of good quality and in adequate quantities in schools, as a key strategy to optimize nutrition of children -calls for restriction of marketing and sale of unhealthy foods in/around schools	Ministry Health/Ministry of Education
	National School Meals and Nutrition Strategy 2017–2022	Strategic Objective 1: To increase awareness and intake of adequate, locally available and nutritious foods among school children and their communities	Ministry of health, Ministry of Education, Science and Technology, Ministry of Agriculture, Livestock and Fisheries
Disease specific policy documents	National guidelines for cancer management, 2013	Objective 2: Reduction of risks posed by unhealthy diets and physical inactivity; advocacy for taxation of sugary drinks	Ministry of Health
Fiscal policies	Excise tax, 2015	Excise tax on soft drinks, including those with or without sugar and sweeteners, at 10 shillings per litre	Ministry of Finance
Legislation	Labeling of Prepackaged Foods – Specification [KS EAS 38:2014]	Provides the specifications for labelling of food products, including ingredient lists/nutrition information panels, as details that should be included in food labels. These standards, developed for individual food categories and items, have been adopted and are implemented by the Kenya Bureau of Standards (KEBS).	Government of Kenya*
	Food, Drugs and Chemical Substances Act Cap 254 (Amendment) 2015	Requires that declaration is on the actual level of trans fatty acids on all foods containing edible fats or oils	Government of Kenya *

*specific contributors to the development of the document are not stated

Respondents said that health policies in Kenya are often shaped by global commitments. Many policies were informed by international policy guidance such as the *Sustainable Development Goals* and the *UN Decade of Action for Nutrition 2016–2025*. In particular, the focus on sugar, salt and fats for NCDs prevention reflected the adoption of the WHO ‘best buys’ [24]. A senior officer from the Ministry of Health highlighted the impact of these commitments at a regional level.

Kenya is member country and anything that is passed by [World Health Organization] WHO as member country, we are obliged to take it up as a country, and also East Africa as well.” KII - representative from the Ministry of Health, 3.

Most of the NCD-related policy documents are developed and implemented by the Ministry of Health, with some involvement from other sectors such as agriculture, education, regulation and standards (such as that necessary to support food labelling). Respondents felt that, although the NCD-related policies exist, their implementation is poorly resourced. One proposed reason for this was that the effects and impact of NCDs take longer to manifest than some infectious diseases.

‘.Priority in terms of policy in books is one thing … but when it comes to resource allocation, that’s a different thing … the infectious diseases have a better leverage on the Ministry of Finance.’ KII with a representative from ministry of health, 1.

In general, there was an emphasis on Government action for treatment rather than prevention of NCDs. Respondents also felt that there was preferential resource allocation for communicable diseases, which contributed further to the unsatisfactory implementation of existing NCDs policies.

‘If you look at the NCDs division it has just a few people – one person per program if I may put it that way. In terms of resources, the Government has not put in enough as compared to let’s say communicable diseases, let’s say HIV, malaria which have full-fledged programs with their own buildings. In terms of that commitment I feel like the Government can do better.’ KII, representative from civil society, 3.

The taxation of SSB is highlighted in the National Cancer Control Strategy [25]. However, it is yet to be implemented in Kenya. Although Kenya has no stand-alone policies on SSB taxation, an excise tax of 10 shillings (0.10 USD) per litre is charged on all soft drinks (with or without sugar and sweeteners), which translates to about 11% per litre of a standard coca cola drink. In 2018, the Financial Bill introduced an excise tax of 20 shillings (0.20 USD) per kilogramme on sugar confectionery and chocolate [26]. Respondents recounted that these taxes were not based on any evidence related to NCDs or SSBs, but were a revenue generation strategy, and that the taxes would likely have a minimal impact on the consumption of SSBs, as most manufacturers absorbed the cost of the taxes to maintain the affordability of their products.

… As you see in the media there was a small increase (tax) in terms of drinks, that time it was considered under drinks, water, juices. I know there was an increase at one time but not as significant to tip (lower consumption) … it was more to increase Government revenue. Of course it has a justification but I don’t think it was sustained on evidence. There is much more we can do from a health perspective”. KII- representative from Ministry of Health, 1.

### Category 3: stakeholder politics related to NR-NCDs and SSB

Several Government ministries are actively engaged with NCD policy implementation. Multi-sectoral policy documents task the national Government with leadership and stewardship roles in addressing NCDs, while the health sector leads the implementation through a dedicated NCD division. This division comprises several sections, including cancer, diabetes, cardiovascular diseases (CVD) and mental health. The Ministry of Agriculture is responsible for food and nutrition security policy. The Ministry of Industry, Trade and Cooperatives coordinates the establishment of standards, including food and beverage standards. The Ministry of Education implements nutrition interventions targeting school children.

Although these sectors are implicated in the same policy documents, they hold different, sometimes opposing, interests. For example, while the Ministry of Health is interested in improving health and discouraging the trade, production and marketing of unhealthy foods, the Ministry of Industry, Trade and Cooperatives promotes the sugar and food processing industries as major revenue sources for the Government. For instance, to enhance the growth of the sugar industry, the Government waived all debts owed to it by the sugar companies including tax penalties and related interests, in 2009 [20]. The national *Big 4 Agenda* also promotes industrialisation, as one of the four pillars of national development, and the food and beverage industry is one of the major industries [27]. Respondents felt that the opposing priorities of Government departments meant that public pressure was integral to improving SSB-related taxation policies. They emphasised the need for the Government to improve financial resources to support NCD prevention activities, such as awareness creation, and screening and health systems strengthening.

It is better if the SSB taxation agenda is seen as an agenda driven by the public than a Government internal process by the Ministry of Health because within the same Government, the Ministry of Industrialization [Ministry of Industry, Trade and Cooperatives] mandate is to promote industry so it becomes a policy conflict (taxation of SSB) for it to be coming from us (Ministry of health).” KII- representative from the Ministry of Health, 1.

Participants also identified a need for local and regional or international evidence to inform and guide decisions on the development and implementation of SSB tax-related policies; specifically, evidence on the economic costs of SSB taxation, the impact of SSB taxation on macro and micro economies of the country, and the social-cultural practices that lead to higher consumption of SSBs.

… we need more evidence and packaging of that evidence that can influence policy, and exactly how the taxation will take place and, what the benefits and the problems that may be encountered in such taxation.” KII- representative from civil society, 1.

Respondents viewed the role of civil society as advocating and supporting the Government with regard to policy development and implementation of NCD prevention and care programmes. The Kenyan NCD Alliance focuses on NCD prevention and coordinates action with other civil society organisations (CSOs). Respondents reported that the NCD Alliance and the Ministry of Health lobbied for SSB taxation in 2016. At the time of this study, this action had not resulted in any tax reform. Respondents thought that the lack of national consensus on the risk of sugar and sugary beverages to support the implementation of SSB tax was a significant barrier to progress.

Unlike tobacco which has a legal framework, [and is recognised as unhealthy] … SSB is part of food and it becomes a bit tricky to criminalise it. Policy maker’s right across parliamentarians, Government workers and all civil society are in so much agreement about tobacco. But when it comes to sugar it is reduced to [consumer] choice.” KII-representative from the Ministry of Health, 1.

Industry tactics and lobbying against SSB taxation were viewed by respondents as major barriers to its eventual adoption. The major SSB companies were described as being huge and powerful, in terms of both their operations, and their contributions to Government revenue and the economy. Respondents described aggressive advertising strategies in the media and on billboards, and education and sports’ sponsorships, and the formation of industry alliances to fight increase in taxation of their products.

As I told you, the Kenya Association of Manufactures come out guns blazing whenever any of the clients is subjected to scrutiny in terms of ‘we need to tax ‘or ‘what is the health of this product’.” KII -representative from the Ministry of Health, 1.

Some elements of industry were also said to have a level of influence over Government decision-making. Key policy and decision makers in the country are shareholders of sugar and SSB companies, which creates a conflict of interest and a challenge for policy actors who are overwhelmed by the industry’s extensive resources.

… .unfortunately there’s a lot of industry interference with policy [on SSB]. This is a big industry; very big in terms of capital and also in terms of influence. They pay a lot of tax to Government and they have a lot of leverage and though the issue here again is not only a Ministry of Health issue is an industrialization issue and also a manufacturing practice issue … an industry like that of course has a lot of policy interference because they have big money they can compete with us.” KII - representative from the Ministry of Health, 1.

## Discussion

NR-NCDs are gaining recognition and there are Government efforts to identify and implement effective interventions to prevent and control them. The findings from this study are consistent with those from a 2014 policy review [28]. However, concerns about the slow progress in the implementation of the policies were evident, with the main hurdle being poor allocation of resources to address NCDs. Other studies have documented poor funding as a challenges in the implementation of NCDs policies in Kenya [29], and have reported that the health sector in Kenya is curative rather than preventive-focused [29]. Similar to this study, Anyona et al. (2014), observed the preference of infectious diseases over NCDs in resource allocation in Kenya, and posited that the non-immediate impact of NCD interventions was less appealing politically as infectious diseases had quick visible results [28]. Policies that enhance equitable access to both preventive and curative healthcare are recommended to address NCDs [28,30].

Although SSB taxation is recommended as a cost effective intervention to prevent NCDs [9], the existing excise tax on beverages does not differentiate between healthy and unhealthy beverages. The excise tax is also not based on any public health evidence; is primarily a revenue collection strategy. No local impact case for SSB taxation has been presented to the Kenyan Government, to demonstrate the potential gains of SSB tax. Preparing this case is especially difficult, given that information about the beverages industry is opaque. Consumption and sales data about soft drinks was not accessible to us as researchers. Although Kenya is collecting NCD data, these data have not been adequately applied to advocate for SSB taxation.

The tobacco taxation policy was introduced in Kenya in 2014, and lessons learned during that process may be applicable to SSB taxation, especially with regard to understanding industry strategy and influence, the need for continuous stakeholder engagement and advocacy, adequate resource allocation, and political leadership and coordination mechanisms [14]. An additional hurdle in the implementation of an SSB tax is the lack of public information about the ill health effects of SSBs, especially in urban communities that place an aspirational value on western diets. Both policy makers and the general public require more information on the health impacts of SSBs and their contribution to NR-NCDs.

Advocacy and public education about NR-NCDs are critical if the current excise tax on soft drinks is to change to SSB taxation. Public education on the health impact of SSB consumption could be done through existing structures such as the education system, the community health structure and the media. The existing stakeholders and the policy framework provide a platform for this work. Local investment in the sugar and SSB industry should be critically examined to understand how this may undermine the Government’s commitment to addressing NCDs through regulations limiting the production and consumption of unhealthy foods and beverages. Furthermore, there is need for additional evidence to support an explicit SSBs taxation policy and wide stakeholder engagement, especially that of the Ministry of Agriculture, Trade and Industrialisation, which are driving the agenda for the sugar and SSB industries in Kenya.

Lack of local evidence on the potential impact of SSB tax was cited as a challenge to lobbying for SSB taxation in Kenya. Researchers should focus on understanding the potential impact of the existing excise tax on SSBs and sugar consumption, and public health, thereby generating ‘practice-based evidence’. However, as consensus is being strengthened around SSB taxation, the existing excise tax creates a window of opportunity for sustained advocacy to gradually increase the tax as more evidence on the impact of SSB consumption is generated.

## Limitations

We targeted to interviewed representatives from various institutions, including the Government, non-governmental organisations, and industries associations. However, industry representatives who were requested to participate in the study were not forthcoming. In addition, obtaining data from the sugar and SSB industry was a challenge; information on industry operations was not readily available on the companies’ websites and formal data requests to the industries were not successful. The findings of this study are therefore limited by the data that were publicly available and accessible at that time of the study.

## Conclusion

The current policy landscape for NR-NCD prevention in Kenya provides some basis for the adoption of an SSB tax, although this is not an explicit policy of Government. NR-NCDs prevention policies should reflect a continuum of issues, from undernutrition to food security, nutrition transition, and the escalation of nutrition related non-communicable diseases. A local advocacy case for sugar-sweetened beverage taxation has not been made, to do this, data such as the cost of illness associated with SSBs and the cost of inaction is needed. Public and policy maker education is critical to challenge the prevailing attitudes towards sugar-sweetened beverages and the western diet.

## Data Availability

Data are available on request.

## Paper context

NCDs are projected to be the leading cause of death in Africa by 2030. High intake of sugar-sweetened beverages (SSBs) contributes to obesity and nutrition-related NCDs. SSB taxation and policies are hindered by inadequate evidence, competing interests of Government, and industry interference. There is a need to generate and use local evidence in NCD and SSB policy development, leverage on Civil Society Organizations’ support for advocacy on NCD, and enhance public awareness on SSB health risks.
